# Lax Uterosacral Ligament and Urge Urinary Incontinence: MRI Findings in Symptomatic Patients Versus Healthy Volunteers

**DOI:** 10.1007/s00192-023-05722-y

**Published:** 2024-01-19

**Authors:** Rania F. El-Sayed, Noha M. Moqbel, Ahmed F. Hussein, Mohamed A. Abdelatty, Sameh A. Hanna, Mohamed S. Abdel Azim

**Affiliations:** 1 Department of Diagnostic and Interventional Radiology, Kasr Al-Ainy Hospital, Cairo University, Kasr Al-Ainy Street, Cairo, 11956 Egypt; 2 Department of Urology, Kasr Al-Ainy Hospital, Cairo University, Kasr Al-Ainy Street, Cairo, 11956 Egypt

**Keywords:** Urge urinary incontinence, Magnetic resonance imaging, Uterosacral ligament

## Abstract

**Introduction and hypothesis:**

The objective was to explore the association between urge urinary incontinence (UUI) and lax uterosacral ligaments (USL) using MRI.

**Methods:**

Sixty-seven female participants were recruited prospectively: 41 continent volunteers (control group) and 26 patients with UUI. Static proton density- and T2-weighted turbo spin echo sequences of MR images were used. A radiologist employed a standardized grid system to record structural observations of the USLs on sequentially numbered axial MR images and then applied a four-point grading scale to assess ligament visibility. MR images were interpreted by a radiologist and a urologist, and then validated by an expert radiologist.

**Results:**

The comparison between the mean length of uterosacral ligaments in the control and UUI groups was highly statistically significant (*p* < 0.001). The mean length of the right USL was 38 ± 11 mm, and the left USL was 35 ± 12 mm in the UUI group. In the control group, the mean length of the USL was 22 ± 9 mm on the right side and 18 ± 9 mm on the left side, along their craniocaudal extent. The highest inter-observer agreement was on the level of origin and insertion (image numbers), whereas the lowest agreement was on the anatomical site of origin and insertion of the USL in both the control and UUI groups.

**Conclusions:**

The average length of USLs in patients with UUI is significantly longer than that in healthy continent women, indicating laxity. Our findings support the relationship between the laxity of the USL and UUI symptoms and have therapeutic implications.

**Supplementary information:**

The online version contains supplementary material available at 10.1007/s00192-023-05722-y

## Introduction

Urge urinary incontinence (UUI) refers to the involuntary leakage of urine associated with a sudden urge to urinate and the inability to reach the lavatory in time [1]. Owing to its unpredictable nature, UUI significantly impacts the quality of life and has a prevalence ranging from 25 to 29% [2].

The management of UUI includes pharmacological treatments [3] and minimally invasive interventions such as posterior tibial nerve stimulation and Botox injections [4, 5]. However, the cure rates for the latter two treatments have been reported to be "unsatisfactory," not exceeding 30–40%, with short-term effects that necessitate repeated treatments [4, 5]. In rare cases, surgical interventions, such as bladder augmentation or urinary diversions, may be required for refractory UUI cases [4–6].

The precise etiology of UUI remains unknown, and various theories have been proposed to explain its pathophysiology in women. In 1993, Petros and Ulmsten suggested that lax uterosacral ligaments (USLs) might play a crucial role in causing UUI by compromising the ability of the vaginal membrane to support bladder stretch receptors [7, 8]. In 2010, Petros and Richardson explored the potential surgical curability of UUI symptoms by performing surgical operations that mimicked the action of the USLs using a posterior tissue fixation system, resulting in an improvement rate of 78% [9].

Several studies, such as those conducted by Jäger et al. in 2012 and Ludwig et al. in 2016, followed the integral theory and employed different surgical approaches that involved the replacement of USLs with alloplastic tapes, yielding favorable results [10, 11]. However, none of these studies included an imaging modality to document the relationship between lax USLs and UUI.

Only a few studies have described the visibility and appearance of USLs in healthy women using MR imaging [12–14], and a single study was conducted to assess the thickness of USLs in patients with UUI [15]. Consequently, the aim of our study was to radiologically investigate the correlation between lax USLs and UUI preoperatively.

## Patients and Methods

This prospective comparative study was conducted between January 2019 and November 2021 at Cairo University Hospitals in Cairo, Egypt, within the departments of diagnostic radiology and urology. The study received approval from the Institutional Review Board, and written consent was obtained from all participants.

### Recruitment of Volunteers

Volunteers were targeted among hospital employees through a local advertising campaign within the hospital premises. Informative advertisements were placed in common areas and on staff notice boards. Hospital employees with no pelvic floor symptoms were invited to contact the research team, and those expressing interest were provided with detailed information about the study's goals, procedures, and benefits, including urological consultation. All participants underwent assessment using a validated questionnaire [16]. Subsequently, 41 healthy continent women were identified and included.

### Symptomatic Participants

Women with urinary incontinence were referred from the Urology department. Patients were evaluated by a urologist (A.H.) with 8 years’ experience, including clinical history, physical examination, laboratory investigations, and urodynamics. Patients with stress and mixed urinary incontinence were excluded, as well as claustrophobic and pregnant patients. Accordingly, 26 patients with UUI were enrolled in the study.

### Magnetic Resonance Imaging

A 1.5-T MRI unit (Achieva; Philips Medical Systems, Best, the Netherlands) with a phased array coil was used. Patients were positioned supine with elevated knees. Prior to the MRI examination, patients were advised to use a rectal cleaning enema and void their bladder 2 h in advance. The rectum was filled with ultrasound gel, and patients were instructed to empty it entirely. MR defecography was performed in the sagittal plane using BFFE (TE/TR: 1.27–1.88/3.3–397.4), slice thickness 8 mm, FOV 250–310, and matrix 512 × 512, with an acquisition time of 2.12 min for each sequence. Dedicated static images of the USLs were acquired using proton density (PD)- and T2-weighted turbo spin-echo sequences in the axial plane (field of view 200 mm, slice thickness 4 mm; gap, 0.5; matrix 512 × 512, acquisition time 2.12 min for each sequence).

### Image Analysis

Magnetic resonance imaging data sets were assessed by a trained radiologist (N.M.), a trained urologist (A.H.), and an expert radiologist (R.E.) with 25 years of experience in pelvic floor MRI. All measurements were obtained using software (Paxera Viewer, V 8.1.7). The expert radiologist remained blinded to the clinical data of all the included participants. Accordingly, the average length of the USLs was based on the analysis of the expert reader.

### Analysis of MR Defecography Images

Pelvic organ prolapse (POP) was evaluated in the MR defecography midsagittal plane, with the pubococcygeal line serving as the reference line. Staging of POP in all three compartments was achieved by measuring the perpendicular distance from the reference line to the anatomical reference point of each compartment [17].

### Analysis of Static MR Images

**Fig. 1:**
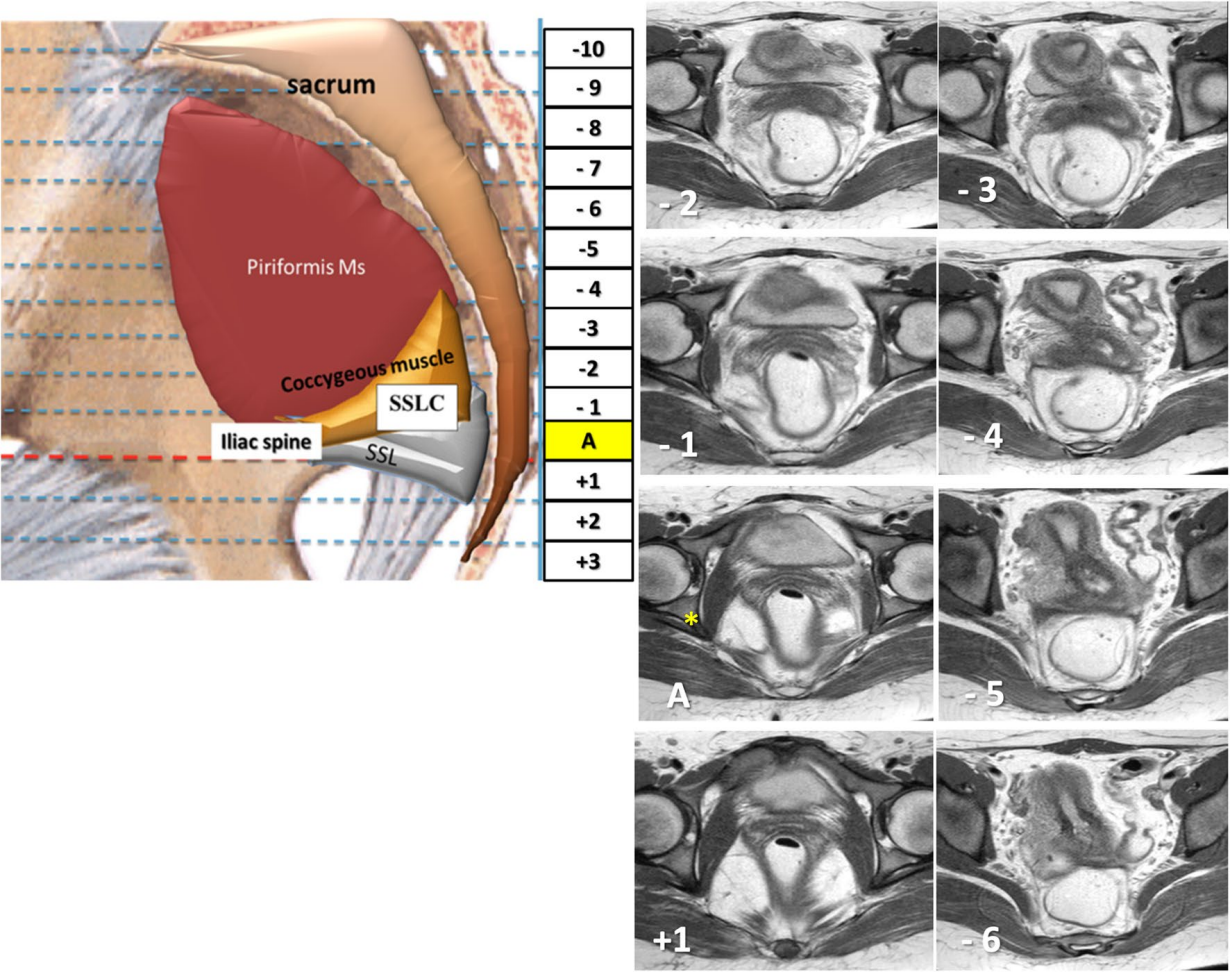
Grid system for uterosacral ligament evaluation: sequential MRI axial proton density-weighted images of a 25-year-old woman with urge urinary incontinence; image “A” was chosen to be the reference level and shows the tip of the ischial spine (*asterisk*); sequential images cephalad to image “A” were denoted with negative numbers and those caudad with positive numbers. *SSL* sacrospinous ligament, *SSLC* sacrospinous ligament-coccygeus muscle complex

Analysis of static MR images involved evaluating the USLs to determine their site of origin from the genital tract and their insertion site on the pelvic sidewall in healthy volunteers, with comparisons made with women with UUI. A standardized grid system, similar to that described by Chou and DeLancey in 2001 and El Sayed et al. in 2007, was used to record structural observations of the USLs on sequential axial T2- and PD-weighted images [18, 19]. Cumulative data from the volunteers and patients were compiled so that the appearance and location of each ligament could be defined. The grid system utilized the tip of the ischial spine as the reference level, labeled "image A." Sequential images cephalad to image "A" were denoted with negative numbers, and those caudad with positive numbers (Fig. 1).

**Fig. 2:**
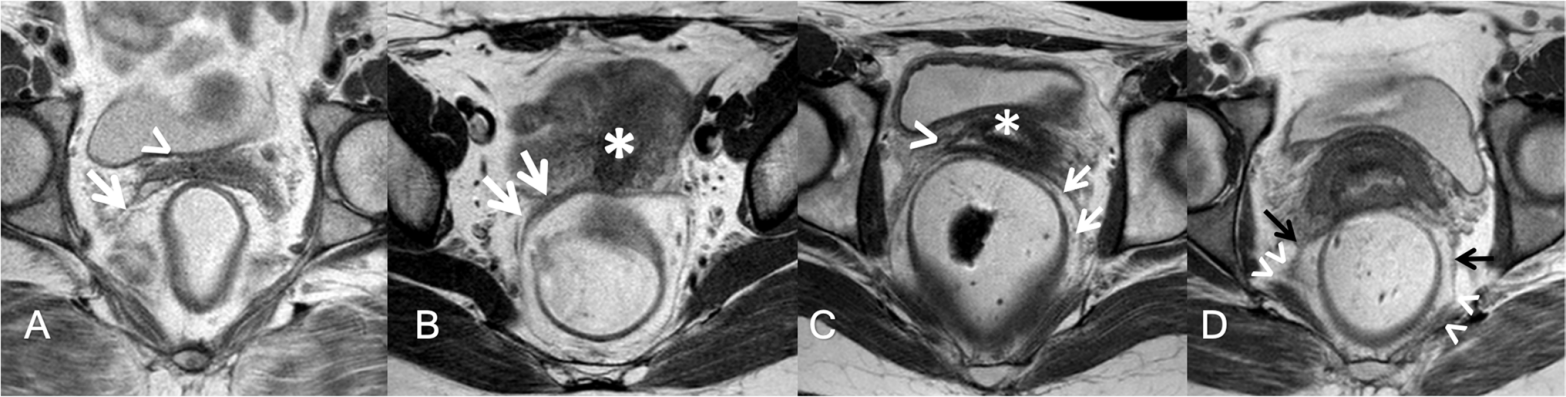
Determination of spectrum of origin and most frequent site of insertion of uterosacral ligaments (*USLs*): representative axial proton density-weighted images of four different women illustrating the different USL origin points: **A** A 56-year-old female volunteer showing the origin of USLs (*white arrows*) from the vagina (*arrowhead*). **B** A 38-year-old female volunteer showing the origin of USLs (*white arrows*) from the cervix (*asterisk*). **C** A 25-year-old female volunteer showing the origin of USLs (*white arrows*) from both the vagina (*arrowhead*) and the cervix “*asterisk*.” **D** A 42-year-old female volunteer showing USLs (*black arrows*) insertions at the sacrospinous ligament-coccygeus muscle complex (*arrowheads*)

The "origin" of the USL was defined as the caudal-most point where the connective tissue condensed into a band-like structure attached to the genital tract in the vagina, cervix, or both. This condensation of connective tissue (USL) had to be visible on at least one image. Similarly, the USL insertion point on the pelvic sidewall was determined according to the structure where the ligament terminated (Fig. 2).

**Fig. 3:**
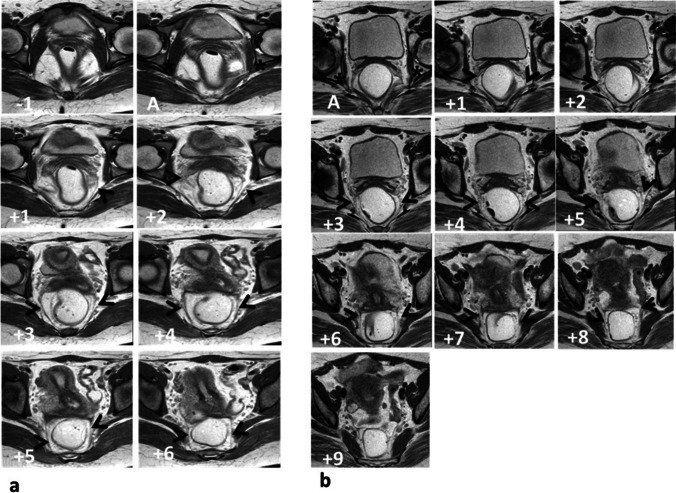
Static MR axial proton density-weighted images: demonstrating the application of the grid system. Image “A” was chosen to be the reference level and showing the tip of the ischial spine (*asterisk*); sequential images cephalad to image “A” were denoted with positive numbers and those caudad with negative numbers. **a** A 23-year-old control female showing USL origin from both the vagina and the cervix (on both sides), uterosacral ligament (*USL*) insertion (sacrospinous ligament and sacrum on both sides). Both USLs are seen (from slice No. +2 to +6 on the right side, and from +1 to +6 on the left side), USL length right side 5 × 4.5 = 22.5 mm and left side: 6 × 4.5 = 27 mm. **b** A 39-year-old woman with urge urinary incontinence showing USL origin: from the vagina (on both sides), USL insertion sacrospinous ligament coccygeus muscle complex and piriformis muscles (both sides). Both USLs are seen (from slice A to +8 on both sides), USL length on both sides 9 × 4.5 = 40.5 mm

According to the grid system, the images on which the origin and insertion of each USL could be determined were reported on both axial T2- and PD-weighted images in relation to image "A." The number of slices on which each USL was visible was counted to determine the length of the USLs. The length of each USL was calculated by multiplying the number of these slices by the sum of each slice thickness and gap (Fig. 3).

**Fig. 4:**
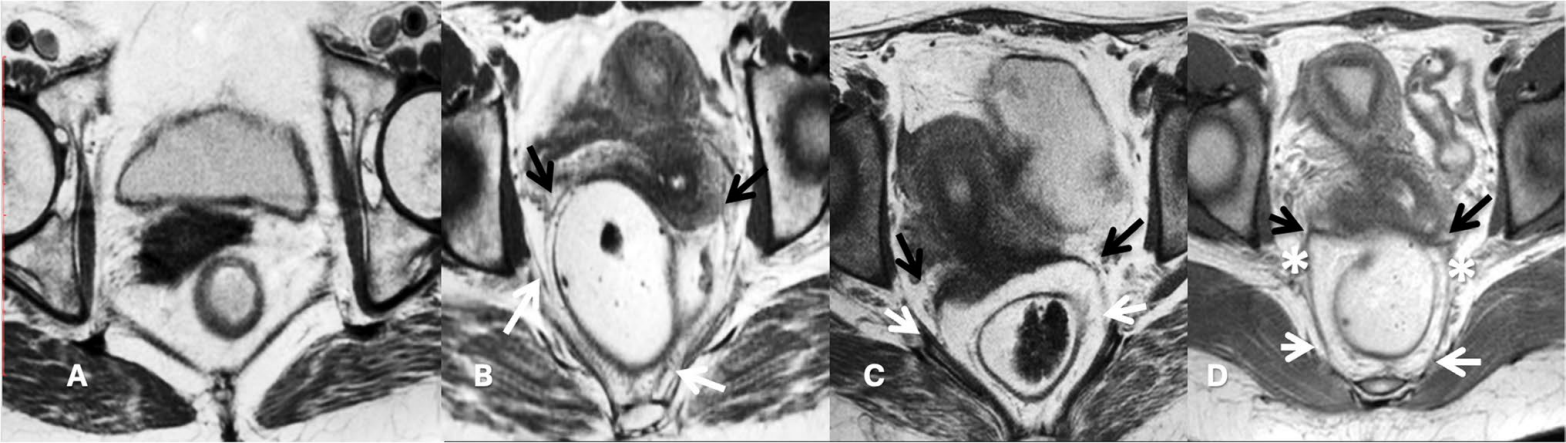
Visibility score of uterosacral ligaments (*USLs*): axial proton density weighted images from four different volunteers showing examples of the visibility score of USLs. *Black arrows* represent the origin, *white arrows* point at the insertions and *asterisk* along the course. **A** A 64-year-old volunteer showing a visibility score 1 = not visible. **B** A 74-year-old volunteer showing a visibility score 2 = poorly visible. **C** A 23-year-old volunteer where the visibility score was 3 = moderately visible. **D** A 25-year-old volunteer with a visibility score of 4 = easily visible

The visibility of USLs was scored using a four-point grading scale, ranging from 1, not visible, 2, poorly visible, 3, moderately visible, to 4, easily visible (Fig. 4). The visibility of the ligaments was further compared on T2- and PD-weighted images.

### Interobserver Reliability

Interobserver reliability was assessed among the three observers for the site of the USLs' origin from the genital tracts, the site of insertion into the pelvic side wall, the slice number on which these anatomical attachments were detected, and the visibility score.

### Statistical Analysis

Statistical analysis was performed using Statistical Package for Social Sciences version 22.0 (SPSS). Numerical data were statistically described in terms of mean ± standard deviation (± SD), whereas categorical data were described in frequency and percentages. Numerical data were tested for the normal assumption using Shapiro–Wilk test. Comparison between the study groups was done using Student’s *t* test for independent samples in comparing normally distributed data, and the Mann–Whitney *U* test for independent samples for comparing non-normal data. Bonferroni adjustment of multiple comparisons was applied when appropriate. To compare categorical data, the Chi-squared test was performed. The exact test was used instead when the expected frequency was less than 5. The kappa statistic was used as a measure of agreement beyond chance. Fleiss Kappa's measure of agreement tested the agreement between categorical variables, with the value of kappa (κ) indicating the strength of agreement. Kappa statistics were interpreted as follows: 0.01–0.20 (slight agreement), 0.21–0.40 (fair agreement), 0.41–0.60 (moderate agreement), 0.61–0.80 (substantial agreement), and 0.81–0.99 (almost perfect agreement). For continuous data Cronbach's alpha was used as a reliability measure with the same concept as kappa. Two-sided *p* values less than 0.05 were considered statistically significant.

## Results

### Study Population

The study included 67 female participants, comprising 41 healthy continent volunteers with a mean age of 38 years (range 15—74 years), and a mean parity of 2.67 (range 0–6). Among them, 7 (17%) were nulliparous, 29 (70.7%) had had previous vaginal deliveries, and 5 (12%) had had previous Cesarean sections. The UUI group included 26 women with a mean age of 37 years (range 15–68 years) and a mean parity of 3.9 ± 1.9 (range 0–8). In this group, 6 patients (23%) were nulliparous, 17 patients (65%) had had previous vaginal deliveries, and 3 patients (11.5%) had had previous Cesarean sections. The control group was further divided into two subgroups based on their MRI defecography findings: group A (*n* = 27) exhibited no pelvic organ descent, and group B (*n* = 14) demonstrated POP below the pubococcygeal line. Similarly, UUI patients were classified into two subgroups: group U1 (*n* = 17) with UUI without POP and group U2 (*n* = 9) with UUI and POP.

### Evaluation of POP

Regarding the severity of POP among subgroup B of the control group, the three-compartment approach was followed and graded according to the rule of three. In the anterior compartment, 7 participants (50%) demonstrated grade I cystocele, whereas 3 participants of this group (21%) showed grade II cystocele. In the middle compartment, 4 participants (28.5%) showed grade I uterine prolapse and 1 (7%) showed grade II uterine prolapse. In the posterior compartment, most participants in this group (10; 71%) showed grade II anorectal junction (ARJ) descent, whereas 3 (21%) showed grade III ARJ descent and 1 (7%) showed grade I ARJ descent. Additionally, 7 (50%) showed grade I rectocele, 5 (36%) showed grade II rectocele, and only 1 (7%) showed grade III rectocele.

Among subgroup U2 (UUI and POP), the severity of POP was as follows, regarding the anterior compartment: 4 participants (44%) demonstrated grade I cystocele, whereas 2 participants (22%) of this group showed grade II cystocele. In the middle compartment, 5 participants (56%) showed grade I uterine prolapse and 1 (11%) showed grade II uterine prolapse. In the posterior compartment, most participants (7; 78%) also showed grade II ARJ descent, whereas 2 (22%) showed grade III ARJ descent. Additionally, 3 (33%) showed grade I rectocele, 3 (33%) showed grade II rectocele, and only 1 (11%) showed grade III rectocele.

### Evaluation of USL Length

Comparison of the mean length of USLs in the control group and in the UUI group revealed a highly statistically significant difference (*p* < 0.001). In the patient group, the mean length of the right USL was 38 ± 11 mm, and the left USL was 35 ± 12 mm along their craniocaudal extents. In contrast, the control group had a mean length of 22 ± 9 mm for the right USL and 20 ± 9 mm for the left USL. A detailed comparison was conducted between the control and patient subgroups, with the results summarized in Table 1. Significant differences were detected between subgroup U2 and control subgroup B, whereas no statistically significant difference was observed between subgroups U1 and U2 concerning the length of the USL on both sides.

**Table 1:** Comparison of uterosacral ligament (*USL*) length between control and urge urinary incontinence (*UUI*) patients and sub-groups based on the expert radiologist measurements on the proton density-weighted MR images

**Control** (*n* = 41)	**UUI patients** (*n* = 26)	*p* value
Right: 22 ± 9 mm	Right: 38 ± 11 mm	< 0.001**
Left: 18 ± 9 mm	Left: 35 ± 12 mm	< 0.001**
**Control** (*n* = 41)	**U1** (*n* = 17)	
Right: 22 ± 9 mm	Right: 40 ± 14 mm	0.0000005**
Left: 18 ± 9 mm	Left: 36 ± 1.4 mm	0.000002**
**Control **(*n* = 41)	**U2** (*n* = 9)	
Right: 22 ± 9 mm	Right: 32 ± 9 mm	0.0001**
Left: 18 ± 9 mm	Left: 32 ± 9 mm	0.01*
**Control subgroup A** (*n* = 27)	**U1** (*n* = 17)	
Right: 22 ± 10 mm	Right: 40 ± 14 mm	0.0001**
Left: 21 ± 9 mm	Left: 36 ± 1 mm	0.002**
**Control subgroup B **(*n* = 14)	**U2 **(*n* = 9)	
Right: 19 ± 7 mm	Right: 32 ± 9 mm	0.02*
Left: 19 ± 7 mm	Left: 32 ± 9 mm	0.02*
**U1** (*n* = 17)	**U2** (*n* = 9)	
Right: 40 ± 14 mm	Right: 32 ± 9 mm	0.19
Left: 36 ± 1.4 mm	Left: 32 ± 9 mm	0.23

Patient subgroup U1 = UUI with negative POP, subgroup U2 = UUI with positive POP; control subgroup B = control with positive POP

*p < 0.05 was considered significant

**p < 0.01 was considered highly significant

### Assessment of USL Origin and Insertion

Regarding the origin and insertion of USLs, the most prevalent site of origin in the control group was the cervix and vagina, whereas in the patient group, it was the vagina. In both groups, the most frequent site of insertion was the sacrospinous ligament-coccygeus muscle complex (SSLC; Table 2). There was no statistically significant difference in the visibility score between T2- and PD-weighted images, as shown in Table 3 in the supplementary material.

**Table 2:** Features of uterosacral ligaments (*USLs*)

	Control				UUI patients			
	PD-weighted images		T2-weighted images		PD-weighted images		T2-weighted images	
	Right	Left	Right	Left	Right	Left	Right	Left
Most frequent site of origin of USL	Cervix & vagina (76%)	Cervix & vagina (73%)	Cervix and vagina (61%)	Cervix & vagina (69%)	Vagina (48%)	Vagina (44%)	Vagina (45%)	Vagina (36%)
Most frequent site of insertion of USL	SSLC (69%)	SSLC (53%)	SSLC (72%)	SSLC (52%)	SSLC (56%)	SSLC (43%)	SSLC (55%)	SSLC (52%)
Image number on which the origin of the USL could be visualized	-2, -3	-2, -3	-1, -2, -3	-1, -2	A, -1, -2	A, -1, -2	A, -1, -2, -3	A, -1, -2, -3
Image number on which the insertion of the USL could be visualized	-5, -6, -7	-5, -6, -7	-5, -6, -7	-5, -6, -7	-6, -7, -8	-5, -6, -7, -8	-6, -7, -8, -9	-6, -7, -8

*UUI* urge urinary incontinence, *PD* proton density, *SSLC* sacrospinous ligament-coccygeus muscle complex

### Interobserver Reliability

For interobserver reliability, the highest agreement among the three readers was found for identifying the slice number where the origin and insertion of the ligament could be located. In the control group, the numbers of the slices were −1, −2, and −3 for the origin, whereas in the patients, the numbers of the slices were A, −1, and −2. Regarding insertion, in the control group, it could be identified in slices numbered −5, −6, and −7, and in the patients, the slices' numbers were −6, −7, and −8. The poorest agreement was observed in assessing the visibility score of USLs (Tables 4 and 5 in the Supplementary material).

## Discussion

Urge urinary incontinence is commonly associated with abnormal detrusor muscle function or innervations [19]. However, recent studies have proposed alternative hypotheses to explain UUI and explored surgical management for potential cures. Notably, in 2012, Jäger and colleagues hypothesized that UUI might be attributed to the laxity of USLs [10, 20]. In our study, we observed a highly significant difference in the mean length of USLs in patients (right USL = 38 ± 11 mm, left USL = 35 ± 12 mm) compared with the control group (right USL = 22 ± 9 mm, left USL = 18 ± 9 mm), indicating significantly longer ligaments in the patient group. These findings align with previous suggestions that USL laxity might be linked to UUI [10, 11].

The comparison between our control and patient subgroups further reinforced this hypothesis. Both subgroups (control B) and (patient U2) exhibited pelvic organ prolapse (POP), yet there was a significant difference in USL length, with longer ligaments in subgroup U2. In subgroup B, the mean length of USLs on the right was 19 ± 7 mm, and on the left it was 19 ± 7 mm. In contrast, subgroup U2 had right USLs measuring 32 ± 9 mm and left USLs measuring 32 ± 9 mm. This suggests that an increase in ligament length could correlate with UUI. In contrast, comparing patient subgroup U1 with U2 revealed no significant difference in terms of USL length (U1 had a mean right USL length of 40 ± 14 mm and left USL length of 36 ± 1 mm) despite the presence of POP. Similarly, comparison between control subgroup A and subgroup U1, both showing no evidence of POP, was statistically significant, with longer USL length in the U1 group. These findings support the idea that POP might not be associated with longer USLs and underscore the strong contribution of USL laxity to the pathogenesis of UUI.

Our decision to employ a grid system for analyzing USL features was aimed at facilitating the differentiation between normal anatomy and occasional anatomical variants, as well as to estimate ligament length. The mean length of USLs in the control group in our study closely matched measurements reported by Umek et al. in 2004 and Chen et al. in 2013, with a slightly longer average length of the right USL than the left USL [12, 14]. This anatomical variation may be explained by the presence of the sigmoid colon and its mesentery in the left pelvis, which leaves less space for the USL on that side [21, 22].

Our results revealed that the most frequent sites of origin of USLs in control group women were both the vagina and the cervix (76% on the right and 73% on the left). This concurs with data from Umek et al. in 2004, where 63% of 61 women had similar USL origin points [12]. However, the most frequent site of origin among UUI patients (48% on the right and 44% on the left) was at a lower level (vagina), further suggesting longer USLs in UUI patients.

The most frequent site of insertion of USLs among the control group was the SSLC (72% on the right and 53% on the left). This also aligns with the findings reported by Umek et al. in 2004, where the USL anatomical insertion point was the SSLC complex in 82% of cases [12, 23]. Our results also showed that the most frequent site of insertion of USLs in UUI patients was the SSLC (56% on the right and 52% on the left).

Using the grid system, we observed that the slice number (level) where the origin of USLs could be seen was most frequently at a lower level among UUI patients than among control participants, whereas the insertion of the USL was found most frequently at a higher level. This further supports the relationship between increased USL length and laxity among symptomatic patients and suggests that USL length might be a contributing factor to the development of UUI.

We found no significant statistical difference in the visibility score between T2- and PD-weighted images, suggesting that either sequence could be used effectively for USL evaluation.

Regarding interobserver reliability, our findings indicated acceptable to good agreement on the slice number (levels) of the origin and insertion (control −1, −2, and −3, patient group A, −1 and −2), followed by the number of slices where USL could be identified. This agreement can be attributed to the use of the grid system, which serves as a reliable and standardized method of measurement. However, the poorest agreement was observed in assessing the visibility score of USLs, as this point is subjective and not obtained using the standardized grid system.

To the best of our knowledge, this study is the first to propose, through radiological evidence using MRI, the influence of lax uterosacral ligaments (USL) as a contributing factor to the pathophysiology of UUI. However, we acknowledge that our study has limitations. First, the sample size for the UUI group was relatively small, a result of our rigorous selection process wherein we excluded cases of mixed (stress and urge) incontinence to ensure the reliability of our results. Further research with larger samples is needed to corroborate our findings. Additionally, the grid system may pose a challenge for nontrained radiologists, yet observers with varying levels of experience can still detect the ligament on the most frequently visible images, ensuring the reproducibility of our findings. Another limitation of our study is the lack of surgical data to support our results; nevertheless, our observations were previously suggested in the urology surgical literature. However, further postoperative MRI evaluation is needed to validate surgical outcomes after USL replacement.

Our findings provide support for a direct correlation between increased USL length and UUI, which could enhance our understanding of the pathogenesis of UUI and potentially broaden therapeutic approaches, especially in cases of refractory UUI.

### Supplementary information

Below is the link to the electronic supplementary material.

Supplementary file1 (DOCX 22 KB)
