# Supplementary material for: Lax Uterosacral Ligament and Urge Urinary Incontinence: MRI Findings in Symptomatic Patients Versus Healthy Volunteers
Source: Int Urogynecol J. 2024 Jan 19;35(4):793–801. doi: 10.1007/s00192-023-05722-y (PMC11052867; doi:10.1007/s00192-023-05722-y)
Supplement: Supplementary file 1 — Supplementary file1 (DOCX 22 KB) [file 192_2023_5722_MOESM1_ESM.docx]

| **Table 3: Visibility score in control and patients’ groups** | | | | | | | | |
| --- | --- | --- | --- | --- | --- | --- | --- | --- |
| **Control** | | | | | **UUI Patients** | | | |
|  | **Right** | | **Left** | | **Right** | | **Left** | |
| **Visibility**  **Score*** | **PD WIs** | **T2 WIs** | **PD WIs** | **T2 WIs** | **PD**  **WIs** | **T2 WIs** | **PD WIs** | **T2 WIs** |
| 4 | 33.30% | 25% | 33.3% | 29% | 68% | 61% | 57% | 52% |
| 3 | 33% | 21% | 23% | 20% | 20% | 26% | 17% | 32% |
| 2 | 30% | 44% | 43% | 43% | 4 % | 13% | 17% | 16% |
| 1 | 3% | 8% | 0% | 8% | 8% | 0% | 9% | 0% |
|  | p-value=0.155 | | p-value= 0.361 | | p-value= 0.986 | | p-value =0.639 | |
| *Grades:1 =not visible, 2= poorly visualized, 3=moderately visualized, 4 =easily visible  *p values were considered significant if p <0.05* | | | | | | | | |

| **Table 4: Inter-observer reliability regarding different MRI features of the USLs** | | | | |
| --- | --- | --- | --- | --- |
| **Inter observer reliability** | **PD WIs** | | **T2 WIs** | |
|  | **Control** | **UUI Patients** | **Control** | **UUI Patients** |
| **Site of origin of USL (Fleiss Kappa)** | Right: 0.36 | Right: 0.27 | Right: 0.37 | Right: 0.35 |
|  | Left: 0.18 | Left: 0.48 | Left:0.29 | Left: 0.34 |
| **Site of insertion of USL (Fleiss Kappa)** | Right: 0.25 | Right: 0.13 | Right: 0.37 | Right: 0.35 |
|  | Left:0.15 | Left: 0.1 | Left: 0.29 | Left: 0.34 |
| **Slices’ number on which origin was visualized (Cronbach’s Alpha)** | Right: 0.84 | Right: 0.84 | Right: 0.84 | Right: 0.85 |
|  | Left: 0.84 | Left: 0.91 | Left: 0.74 | Left: 0.85 |
| **Slices’ number on which insertion was visualized (Cronbach’s Alpha)** | Right: 0.90 | Right: 0.91 | Right: 0.72 | Right: 0.85 |
|  | Left: 0.78 | Left: 0.90 | Left: 0.58 | Left:0.77 |
| **Sequential number of slices from which the USL length was calculated (Cronbach’s Alpha)** | Right: 0.71 | Right: 0.73 | Right: 0.44 | Right: 0.75 |
|  | Left: 0.77 | Left: 0.63 | Left: 0.74 | Left: 0.56 |
| *Cronbach’s Alpha Fleiss Kappa*  0.9 Excellent 0.81–0.99 (almost perfect agreement)  >0.8 Good 0.61–0.80 (substantial agreement)  0.7 Acceptable 0.41–0.60 (moderate agreement)  0.6 Questionable 0.21–0.40 (fair agreement)  0.5 Poor 0.01–0.20 (slight agreement)  < 0.5 Unacceptable | | | | |

| **Table 5: agreement on the visibility score using Fleiss kappa and 95% confidence interval on PD MR images between the three raters** | |
| --- | --- |
|  | **Fleiss kappa** |
| **Control group** | Right: 0.2460 |
|  | Left: 0.1961 |
| **UUI group** | Right: -0.0130 |
|  | Left: 0.0324 |
